# From bedside to bench: the missing brick for patients with fungal sepsis

**DOI:** 10.1186/s13054-016-1378-2

**Published:** 2016-06-21

**Authors:** Vincenzo Russotto, Andrea Cortegiani, Santi Maurizio Raineri, Antonino Giarratano

**Affiliations:** Department of Biopathology and Medical Biotechnologies (DIBIMED), Section of Anaesthesia, Analgesia, Intensive Care and Emergency, University Hospital Paolo Giaccone, University of Palermo, Via del Vespro 129, 90127 Palermo, Italy

We read with great interest the article by Spec et al. [1] investigating the immunophenotype of T cells from patients with *Candida* spp. sepsis. This is the first observational study describing the altered immune response of patients with candidemia. The authors included non-neutropenic critically ill patients with candidemia and non-septic controls, and excluded patients with human immunodeficiency virus infection, who had undergone solid or bone marrow transplantation or with other known causes of impaired immune response. The authors hypothesized that their findings may help explain why patients with fungal sepsis show a high mortality despite appropriate antifungal therapy.

In our opinion, the observed T-cell exhaustion associated with candidemia may also contribute to explain the paradox of the evidence published recently about invasive fungal infection (IFI) prevention [2]. From our recently published Cochrane review [3], the use of antifungals in critically ill patients as untargeted treatment was not associated with any survival benefit despite being associated with a 45 % reduction of IFIs. When prophylactic and empiric antifungal treatments were investigated in sub-analyses, a reduction of IFIs was observed only after prophylactic administration of antifungals. We hypothesized that studies investigating empiric treatment, defined as the administration of antifungals in patients with signs/symptoms of infections at risk for IFIs, included patients with a more advanced disease process, leading to lack of any survival benefit from antifungals. We now may also speculate that poor survival of patients despite untargeted antifungal drugs may depend on their immune status. Patients treated with empiric antifungals may show an impaired immune response, which may play a causative role for reduced survival or may represent a marker of advanced disease process.

Implications for future research may include potential additional mechanisms of impaired immunological function to fill the gap between bedside data and pre-clinical findings.

## Abbreviation

IFI, invasive fungal infection
